# A non-destructive DNA sampling technique for herbarium specimens

**DOI:** 10.1371/journal.pone.0183555

**Published:** 2017-08-31

**Authors:** Lara D. Shepherd

**Affiliations:** Museum of New Zealand Te Papa Tongarewa, Wellington, New Zealand; University of Otago, NEW ZEALAND

## Abstract

Herbarium specimens are an important source of DNA for plant research but current sampling methods require the removal of material for DNA extraction. This is undesirable for irreplaceable specimens such as rare species or type material. Here I present the first non-destructive sampling method for extracting DNA from herbarium specimens. DNA was successfully retrieved from robust leaves and/or stems of herbarium specimens up to 73 years old.

## Introduction

Museum collections, including herbarium specimens, are becoming an increasingly popular source of DNA [1]. They are a readily-available source of material for rare, extinct and difficult to obtain taxa and can provide a historical perspective. The sampling for DNA of type specimens for inclusion in phylogenetic analyses is particularly important because it enables species names to be applied with certainty [2]. For example, Chomicki and Renner [3] sequenced DNA from the holotype of watermelon collected in 1773 and found that this specimen is not the species now thought of as watermelon.

Sampling herbarium specimens for DNA is not without its drawbacks. Herbarium specimens typically have low concentrations of DNA, which is typically degraded [4], although new DNA sequencing technologies are overcoming this issue (e.g. [5,6]). Herbarium specimens are also a finite resource. Current DNA extraction methods involve destroying part of the specimen (e.g., removal of a leaf and grinding it up). Such destructive sampling can limit the future use of a specimen by both geneticists and other researchers. Curators, who are tasked with caring for herbarium collections, need to balance specimen preservation with their use for research. To this end DNA extraction methods that minimize damage to specimens are desirable.

Non-destructive DNA extraction methods have been developed for museum specimens of zoological origin including teeth, bones and invertebrates (e.g., [7–9]). However, there appears to be no previously-published non-destructive methods for obtaining DNA from herbarium specimens. Non-destructive DNA extraction methods for zoological specimens typically involve soaking the animal tissue in an extraction buffer in order to leach out DNA. Herbarium samples are usually mounted on paper, making soaking them difficult, and so a different approach is required. Here, I describe and test the first non-destructive method to isolate DNA from herbarium samples, which is based on a method used to extract animal protein from parchment [10].

## Methods

Twenty-two herbarium samples from the herbarium of the Museum of New Zealand Te Papa Tongarewa (WELT) were included in the sampling; these represented 12 species, from three families of flowering plants and two fern families (Table 1). These specimens ranged in age from two to 86 years old. Only species with robust leaves and/or stipes (stems of fern fronds) were selected for sampling because initial trials showed that the use of the eraser caused damage to more delicate plant specimens.

**Table 1:** Details of the herbarium specimens used in this study.

Species	Specimen voucher	Sampling site	Specimen collection date	Amplicon sequenced; bp (longest attempted amplification; bp)/number of successful PCR reactions	GenBank Accession number	DNA concentration (ng/ul)
Aspleniaceae						
*Asplenium bulbiferum*	LRP2536b	Stipe	2003	372 (372)/3	KY946757	0.358
*Asplenium bulbiferum*	P017781	Costae	1989	n/a (215)/0		0.112
*Asplenium bulbiferum*	P015230	Costae	1963	n/a (215)/0		0.378
*Asplenium flaccidum*	LRP2192	Upper lamina	2002	n/a (215)/0		0.170
*Asplenium gracillimum*	LRP2068	Costae plus upper lamina	2002	215 (372)/1	KY946760	0.236
*Asplenium obtusatum*	P025227	Upper lamina	1974	215 (372)/1	KY946759	0.162
*Asplenium obtusatum*	P022102	Upper lamina	1944	372 (372)/3	KY946758	0.126
*Asplenium obtusatum*	P009385/A	Upper lamina	1931	n/a (215)/0		<0.0005
*Asplenium obtusatum*	P018053	Stipe	1991	n/a (215)/0		<0.0005
Blechnaceae						
*Blechnum procerum*	P027862	Upper lamina	2015	358 (358)/3	KY946761	0.130
*Blechnum procerum*	P016430	Upper lamina	1992	n/a (117)/0		<0.0005
Rubiaceae						
*Coprosma foetidissima*	SP088811	Upper lamina	2010	107 (107)/1	KY946762	<0.0005
*Coprosma foetidissima*	SP083707	Lower lamina	1977	107 (107)/1	KY946763	<0.0005
*Coprosma grandifolia*	SP088799	Upper lamina	2010	107 (107)/1	KY946764	<0.0005
*Coprosma robusta*	SP086154	Upper lamina	2008	n/a (107)/0		<0.0005
Asparagaceae						
*Cordyline australis*	SP088834	Upper lamina	2010	n/a (115)/0		<0.0005
Corynocarpaceae						
*Corynocarpus laevigatus*	SP103738	Lower lamina	2010	683 (683)/4	KY946765	0.364
*Corynocarpus laevigatus*	SP094714	Upper lamina	1974	n/a (91)/0		<0.0005
*Corynocarpus laevigatus*	SP007789*	Upper lamina	1950	n/a (91)/0		<0.0005
Araliaceae						
*Pseudopanax crassifolius*	LRP6021	Upper lamina	2009	390 (390)/2	KY946766	<0.0005
*Pseudopanax lessonii*	LRP4728	Upper lamina	2006	n/a (138)/0		<0.0005
*Pseudopanax lessonii*	LRP4952	Upper lamina	2007	138 (390)/1	KY946767	<0.0005

LRP samples are non-accessioned vouchers held at WELT.

Sample marked * had been treated with HgCl_2_.

Amplicon sequenced = maximum length in base pairs (bp) of successful PCR amplification product. n/a = no PCR amplification.

<0.0005 indicates samples that failed to produce a reading with the Qubit and therefore have a DNA concentration lower that the Qubit detectable limit.

Sampling was performed within the herbarium using a Staedtler “Mars Plastic” eraser that had been cut into ~7mm^2^ pieces with a sterile razor blade. The eraser was rubbed in both directions across either the leaf surface or stipe and the resulting eraser fragments (also called erdu) were collected onto paper and placed into a sterile 1.5 ml microcentrifuge tube (S1 Fig). To avoid cross-contamination, new paper and a new piece of eraser was used for each herbarium specimen and gloves were changed between each sample.

Eraser samples were then transferred to an ancient DNA laboratory, where all DNA extractions and PCR set-ups were performed. In order to avoid PCR-product contamination of the ancient DNA laboratory and of the herbarium, PCR amplifications were performed in a modern DNA laboratory located in a different building [11, 12].

DNA extraction was performed with a DNeasy blood and tissue kit (Qiagen, Valencia, CA, USA). The eraser fragments were incubated for 3 hours in Buffer ATL and proteinase-K on a heating block set at 50°C. Following incubation, the manufacturer’s instructions were followed except that the final elution used 35 μl of Buffer AE and was spun through the column twice (the first elution was placed back on the column and spun through a second time). Negative extraction controls containing no eraser fragments were processed in parallel with the sample extractions to monitor for reagent contamination. The sampling and extraction protocol has been deposited on protocols.io (dx.doi.org/10.17504/protocols.io.i3jcgkn).

DNA quality was assessed by examining DNA yield and PCR amplification success. DNA was quantified with a Qubit 3.0 fluorometer using the Qubit dsDNA high sensitivity (HS) assay (Invitrogen). For some taxa, amplification with a number of PCR primers was attempted to test what PCR amplicon length could be obtained. PCR primers and their resulting amplicon lengths are reported in Table 2. Novel chloroplast primers were designed with Primer3 [13] using sequences from the National Center for Biotechnology Information (NCBI) GenBank database. Primers for *Coprosma* and *Asplenium* were designed to target DNA regions whose sequence differs between at least some of the sampled species, based on available sequences from GenBank (*C*. *foetidissima* is distinguished from *C*. *robusta* and *C*. *grandifolia* within the 107 bp amplicon, and all four *Asplenium* species differ within the 215 bp amplicon). This was not possible for *Pseudopanax* because the examined species, *P*. *lessonii* and *P*. *crassifolius*, share chloroplast sequences [14].

**Table 2:** Primers used for testing non-destructive DNA extraction from herbarium specimens.

Target taxon	Forward primer name	Sequence (5’ to 3’)	Reverse primer name	Sequence (5’ to 3’)	Locus, maximum amplicon length
*Asplenium*, Aspleniceae	rbcLAsForward2 [15]	AAGCCAAAATTAGGTCTATCTGC	rbcLAsReverse2 [15]	CCCAATTCTCTCGCAAAAACAG	rbcL, 215 bp
	rbcLAsForward2 [15]	AAGCCAAAATTAGGTCTATCTGC	rbcLAsR3	GCGCGATGAATATGAAGAAG	rbcL, 322 bp
	rbcLAsForward2 [15]	AAGCCAAAATTAGGTCTATCTGC	rbcLAsR4	CACGAAAATGCATACCGTGA	rbcL, 372 bp
*Blechnum*, Blechnaceae	Blechnum_trnLF	GGGGATAGAGGGACTCGAAC	Blechnum_trnLR	CCGGTAGCGGAAAAATGATA	trnLF, 117 bp
	Blechnum_trnLF	GGGGATAGAGGGACTCGAAC	Blechnum_trnLR2	CCAGCATTCATTCACGAAAT	trnLF, 240 bp
	Blechnum_trnLF	GGGGATAGAGGGACTCGAAC	Blechnum_trnLR3	GTGTGGTTAACTGCATGGGATA	trnLF, 358 bp
*Coprosma*, Rubiaceae	CoprosmaF	ACGGGAATCCATCGTTTGTA	CoprosmaR	ACGGTGTTTCCTTGTTTTGG	rps16, 107 bp
*Cordyline*, Asparagaceae	CorAusrbcLF	CAAAGGACGATGCTACCACA	CorAusrbcLR	CTCGTAGGGCTTTGAAACCA	rbcL, 115 bp
*Corynocarpus*, Corynocarpaceae	CorLaerps4F	TTAACTAATTGGCGGGCTTG	CorLaerps4R	GTTCGTATCGCTGGAAAAGC	rps4, 91 bp
	CorLaerps4F	TTAACTAATTGGCGGGCTTG	CorLaerps4Rlong	GTTACAGAGGACCTCGTTTCAAAAA	rps4, 283 bp
	CorLaerps4Flong	CTTTTGGCAATTCCTCATGG	CorLaerps4Rlong	GTTACAGAGGACCTCGTTTCAAAAA	rps4, 435 bp
	CorLaerps4F2long	TACCGATCCACGATACACGA	CorLaerps4Rlong	GTTACAGAGGACCTCGTTTCAAAAA	rps4, 683 bp
*Pseudopanax*, Araliaceae	Pseudorps4F	TCCGTACCTTCATCTAATTCACTG	Pseudorps4R	CGAGAGGGAGGTTTTCATCA	rps4, 138 bp
	Pseudorps4F	TCCGTACCTTCATCTAATTCACTG	Pseudorps4longR	CATTTGACTCTTCGCCCATT	rps4, 390 bp

All primers except rbcLAsForward2 and rbcLAsReverse2 were specifically designed for this study.

Amplicon length excludes primer sequences. bp, base pairs.

All PCRs were performed in 12 μl reactions with 1× Mytaq reagent buffer (Bioline, Australia), 5 ρmol of each primer, 1 M betaine and 15 μg bovine serum albumin. A negative PCR control containing no added DNA was included in each batch of PCRs. Positive DNA controls of modern DNA were added in the modern laboratory to confirm the ability of the novel primers to amplify their targets.

For all amplifications the thermocycling conditions were an initial denaturation of 2 min at 94°C, followed by 35 cycles of 94°C for 30 sec, 50°C for 40 sec and 72°C for 1 min; followed by a final extension of 10 min at 72°C. PCR products were visualized by electrophoresis on a 2% MS/1% LE agarose gel. PCR products were purified by digestion with 1 U shrimp alkaline phosphatase (SAP, USB Corp., Cleveland, USA) and 5 U exonuclease I (Exo I, USB Corp., Cleveland, USA) at 37°C for 30 min, followed by inactivation of the enzymes at 80°C for 15 min. DNA sequencing was performed by capillary separation at the Massey Genome Service (Palmerston North, New Zealand) or Macrogen Inc. (Seoul, Republic of Korea). Sequences were edited in Sequencer 5.2.3 (Gene Codes Corporation) and identification was determined using BLASTn searches of the NCBI GenBank database [16].

## Results

DNA yields were low (Table 1) and did not necessarily indicate whether PCR would be successful. Similarly, specimen age did not appear to predict either DNA yield or PCR success (Table 1). The negative extraction and PCR controls showed no amplification. Half of the 22 specimens tested produced amplifiable DNA for at least the shortest amplicon attempted (Table 1). For specimens that were amplified with multiple primer pairs the resulting sequences were consistent across the different PCR amplifications. All sequences obtained were identical to available GenBank sequences for the same species. The longest DNA sequence generated for each specimen has been deposited in GenBank (GenBank accession numbers are provided in Table 2).

## Discussion

This appears to be the first demonstration of a non-destructive method for the extraction of DNA from herbarium specimens. The results were consistent across multiple PCRs with different primers pairs and the sequences obtained all matched the expected species, indicating that contamination between herbarium sheets was not an issue for this study. Future studies using this method may want to increase the likelihood of obtaining authentic ancient DNA by including negative controls with eraser fragments but no sample, performing the eraser sampling in a cleanroom separate from the herbarium collection area and assessing patterns of DNA damage using next-generation sequencing.

Although chloroplast DNA could be successfully amplified from only half of the specimens sampled, this result is not dissimilar to previous destructive sampling of herbarium specimens (e.g., success rates of 26% (800 bp fragment) to 57% (470 bp fragment) [2]; 10% (670 bp fragments) to 78% (10–143 bp fragments) [17]). In agreement with other studies analyzing the DNA of herbarium material (e.g., [18, 19] but see [2]), age did not seem to predict the quantity of DNA. Specimen preparation (e.g., drying time and temperature, treatment with chemicals) has been suggested to have a greater influence on DNA preservation than age [17, 20] and may explain these results.

Five of the specimens produced amplicons longer than 300 bp but 6 specimens only yielded amplification products shorter than 250 bp (although the maximum amplicon attempted was only 107 bp for 3 of these specimens). Short fragments may be sufficient for identification purposes if mini-barcode primers [17, 21] are used. Alternatively, utilizing next generation sequencing, which targets short fragments of DNA, possibly in combination with target enrichment [22], would enable much larger amounts of sequence to be obtained from DNA extracted using this method.

The successful amplification of DNA from two of the three *Pseudopanax* specimens suggests an additional use of this DNA extraction method. Previous attempts to extract DNA from silica-dried material of *Pseudopanax* were unsuccessful owing to the presence of compounds that impeded DNA extraction [14, 23]. Past genetic studies extracted DNA from fresh leaf material of *Pseudopanax*. It is likely that the eraser technique succeeds because the co-extraction of inhibitory substances are avoided. This method may prove useful for obtaining DNA from other difficult to extract templates or those with co-extracting secondary compounds that inhibit PCR (e.g., [24]).

Caution should be applied when attempting this method. Initial trials on species with more delicate leaves than those examined here caused damage to the leaf surface. Trialing eraser sampling on expendable specimens with leaves of similar robustness to those wanting to be sampled is advisable. For delicate samples, stipes and petioles may provide a more robust source of tissue for sampling than leaves. Future study comparing the success of this method on samples taken from different parts of the same specimen would be useful for determining the optimal regions for sampling.

In conclusion, this study demonstrates that it is possible to non-destructively sample DNA from herbarium specimens. The eraser method is likely to be of particular use for valuable specimens, such as type material and rare or extinct species, where destructive sampling is undesirable

## Supporting information

S1 Fig A. Erdu and eraser. B, C, D. Specimens after sampling for DNA with an eraser. Arrows indicate the sampling sites. The specimens are as follows: B *Corynocarpus laevigatus* SP103738, C *Asplenium bulbiferum* LRP2536b, D *Asplenium obtusatum* P022102.
(PDF)
