# Supplementary material for: A non-destructive DNA sampling technique for herbarium specimens
Source: PLoS One. 2017 Aug 31;12(8):e0183555. doi: 10.1371/journal.pone.0183555 (PMC5578499; doi:10.1371/journal.pone.0183555)

S1 Figure. A. Erdu and eraser. B, C, D Specimens after sampling for DNA with an eraser. Arrows indicate the sampling sites. The specimens are as follows: B *Corynocarpus laevigatus* SP103738, C *Asplenium bulbiferum* LRP2536b, D *Asplenium obtusatum* P022102.

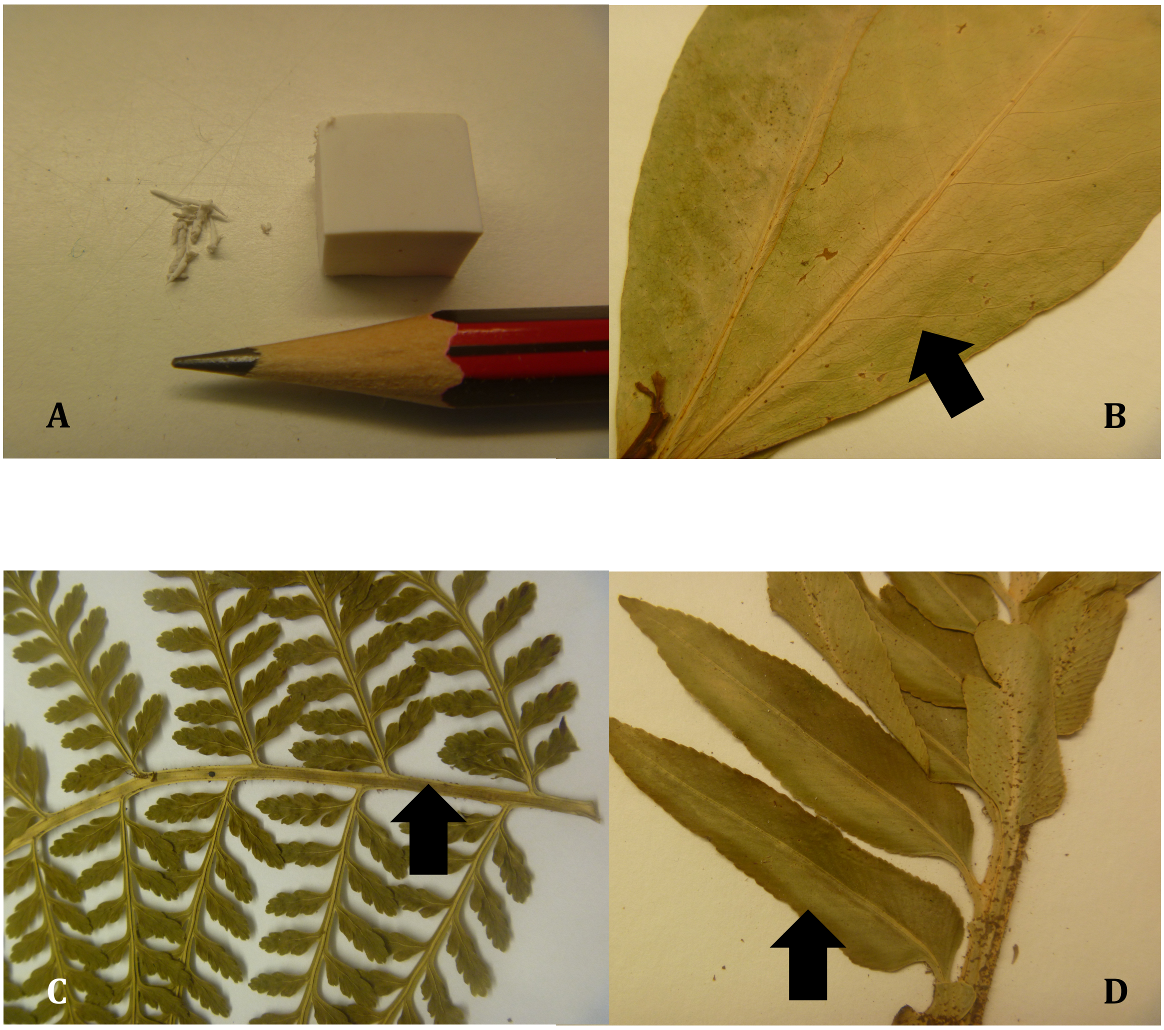

Supplement: S1 Fig — A. Erdu and eraser. B, C, D. Specimens after sampling for DNA with an eraser. Arrows indicate the sampling sites. The specimens are as follows: B Corynocarpus laevigatus SP103738, C Asplenium bulbiferum LRP2536b, D Asplenium obtusatum P022102. (PDF) [file pone.0183555.s001.pdf]
